# New Rickettsiae in ticks collected in territories of the former soviet union.

**DOI:** 10.3201/eid0506.990612

**Published:** 1999

**Authors:** E Rydkina, V Roux, N Rudakov, M Gafarova, I Tarasevich, D Raoult

**Affiliations:** Faculté de Médecine, Université de la Méditerranée, Marseille, France.

## Abstract

Dermacentor nuttallii from Siberia, Rhipicephalus sanguineus from Crimea, and Rh. pumilio from the Astrakhan region were infected with Rickettsia sibirica (12%), R. conorii (8%), and the Astrakhan fever agent (3%), respectively. Three new Rickettsiae of the R. massiliae genogroup were identified in ticks by 16S rDNA, gltA, and ompA sequencing.


### New *Rickettsiae* in Ticks Collected in Territories of the Former Soviet Union

**Elena Rydkina,*† Véronique Roux,* Natalia Fetisova,† Nikolai Rudakov,‡ Mouniver Gafarova,§ Irina Tarasevich,† and Didier Raoult*†**
*Faculté de Médecine, Université de la Méditerranée, Marseille, France;
†Russian Academy of Medical Sciences, Moscow, Russia;
‡Research Institute of Endemic Infectious Diseases, Omsk, Russia; and
§Medical University, Simferopol, Crimea

*Dermacentor nuttallii* from Siberia, *Rhipicephalus sanguineus* from Crimea, and *Rh. pumilio* from the Astrakhan region were infected with *Rickettsia sibirica* (12%), *R. conorii* (8%), and the Astrakhan fever agent (3%), respectively. Three new *Rickettsiae* of the *R. massiliae* genogroup were identified in ticks by 16S rDNA, *gltA,* and *ompA* sequencing.

Rickettsiae are obligate intracellular gram-negative bacteria associated with arthropod vectors, ticks, mites, and insects that, while feeding, can transmit *Rickettsiae* to animals and humans. The rickettsioses have characteristic clinical features, including fever, headache, maculopapular eruption, and sometimes eschar formation (primary lesion). The number of representatives of the genus *Rickettsia* has increased over the last decades as a result of improved cell culture isolation and agent identification techniques (1). Sequence comparison of gene coding for citrate synthase (*gltA*) (2), rOmpA outer membrane protein (*ompA*) (3), and 16S rRNA (4) has become the most reliable method of identifying *Rickettsiae* (5-8). We describe polymerase chain reaction (PCR) amplification and sequence determination to identify *Rickettsiae* in naturally infected ixodid ticks in three regions of Russia endemic for tickborne rickettsioses.

*Rhipicephalus pumilio* ticks (65 adults) were collected in 1996 from dogs in the Astrakhan region. *Dermacentor nuttallii* ticks (101 adults) were collected in 1994 in the village of Verhnyi Kouus, the Altay Mountains, Siberia. In 1997, *Rh. sanguineus* ticks (2 adults and 35 nymphs) were collected in the town of Saki, Crimea region, from dogs whose owners had serologic evidence of Mediterranean spotted fever (Figure 1). The ticks were kept at room temperature before being washed in iodized alcohol (10 minutes) just before testing, rinsed in distilled water, and dried on sterile filter paper. DNA was extracted from ticks by using the QIAmp Tissue Kit (QIAGEN, Hilden, Germany). Rickettsial DNA was detected by PCR with primers specific for *Rickettsiae*: RpCS.877p-RpCS.1273r, which amplify a 396-bp fragment of *gltA* (2), and Rr190.70p-190-701 (3), which amplify a fragment of *ompA* from 629 to 632 bp. For all positive ticks, 587 to 590 bp of *ompA* were sequenced by using the ABI PRISM Dye

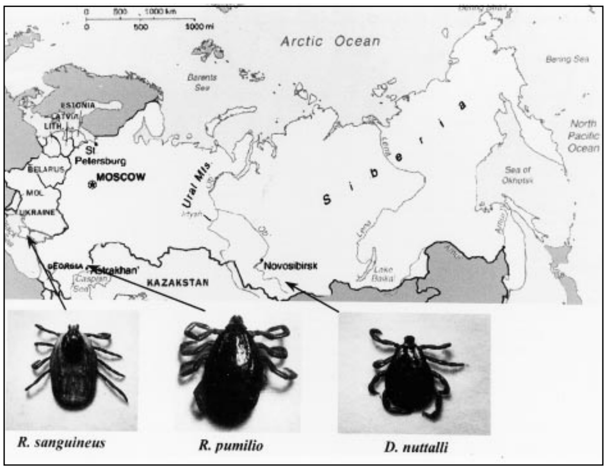

Figure 1. Areas from which ticks in the study were collected.

Address for correspondence: Didier Raoult, Unité des Rickettsies, CNRS UPRES-A 6020, Faculté de Médecine, Université de la Méditerranée, 27 bd. J. Moulin 13385 Marseille, Cedex 05, France; fax: 33-49-183-0390; e-mail: Didier.Raoult@medecine.univ-mrs.fr.

Terminator Cycle Sequencing Kit with Amplitap Polymerase FS (PE Applied Biosystems, Warrington WA1 4SR, UK). Sequences were analyzed with the Applied Biosystem 377 automatic sequencing system. For newly detected genotypes, sequences of 16S rRNA encoding gene, *gltA*, and *ompA* were determined as previously described (2-4) (see Figure 2 for GenBank codes).

We detected two different *Rickettsiae* in *Rh. pumilio* (Astrakhan fever agent and RpA4 genotype); two *Rickettisiae* from *D. nutallii* in Siberia (*Rickettsia sibirica* and DnS14 and DnS 28 genotypes); and *R. conorii* from *Rh. sanguineous* ticks in Crimea (Table).

Our results confirm previous data of high epidemic activity of the Altay focus for North Asian tick typhus and the crucial role of *D. nuttallii* as a reservoir of *R. sibirica* infection (9). Our results are also consistent with those of a study in 1991 based on hemolymph testing (10), in which 3.2% of ticks from the Astrakhan region were demonstrated to be infected with *Rickettsiae*.

An outbreak of Mediterranean spotted fever due to infection with *R. conorii* occurred in Crimea from 1947 to 1957. Only sporadic cases of the disease were reported (11) until 1995, when the incidence of Mediterranean spotted fever increased in central Crimea, with 40 cases in 1996 and more than 70 in 1997. Most cases occurred in the summer, when the *Rh. sanguineus* nymphs (principal vectors of *R. conorii*) (1) were active. Our results, showing that 8% of the *Rh. sanguineus* studied contained *R. conorii* DNA, provide further evidence of the Mediterranean spotted fever outbreak in the region. To date, only the *R. conorii* strain M-1, isolated in the territories of the former Soviet Union (the Black Sea coast of Georgia), has been genetically characterized. This strain is genetically distinct from the other strains of *R. conorii*, i.e., Indian tick typhus and the Moroccan and Malish strains (3). Our detection of the *R. conorii* strain identical to the Malish strain is the first evidence of the genetic heterogeneity of *R. conorii* in the region.

The *ompA* sequences obtained from PCR-amplified products were different from those described for the known *Rickettsiae* for one DNA sample extracted from *Rh. pumilio* from the Astrakhan region (RpA4) and four DNA samples from *D. nuttallii* collected in Siberia (DnS14, DnS28, DnS79, DnS94). The sequences for the samples from *D. nuttalii* (DnS28, DnS79, and DnS94) were identical but differed from those of DnS14 and *Rh. pumilio* RpA4/2.

The three new rickettsial agents were closely related and branched with members of the *R. massiliae* group, together with *R. rhipicephali*, Bar 29, *R. aeschlimannii*, and *R. montanensis* (Figure 2). Comparison of the sequences obtained by using the program BLAST demonstrated that they also differed from those of the Cadiz agent characterized from *Ixodes ricinus* in Spain (6), those of the *Cooleyi* genotype characterized from *I. scapularis* (5), MOA and WB-8-2 isolated from *Amblyomma americanum* and *I. scapularis,* respectively (8), and *R. peacockii* (7) in the United States.

The pathogenicity of the members of the *R. massiliae* group is unknown, and their main reservoirs are regarded as ticks of the genus *Rhipicephalus* for *R. massiliae* and Bar 29. *R. aeschlimannii* has been isolated from *Hyalomma marginatum* and *R. montanensis* from ticks of the genus *Dermacentor*. *R. rhipicephali* has been demonstrated in ticks of the genus *Dermacentor* and in *Rh. sanguineus* (1). The similarity of *gltA*, *ompA*, and 16S rRNA gene sequences indicates that these three new

Table. Ticks infected in regions of the former Soviet Union

| Tick species | Location | No. positive ticks/total examined | % infected ticks | Rickettsial species |
|---|---|---|---|---|
| *Rhipicephalus pumilio* | Astrakhan region | 2/65 | 3 | Astrakhan fever agent |
| *Rh. pumilio* | Astrakhan region | 1/65 | 1.5 | RpA4 genotype |
| *Dermacentor nutallii* | Siberia | 12/101 | 12 | *Rickettsia sibirica* |
| *D. nutallii* | Siberia | 4/101 | 4 | DnS14 and DnS28 genotypes |
| *Rh. sanguineus* | Crimea | 3/37 | 8 | *R. conorii* (Malish strain) |

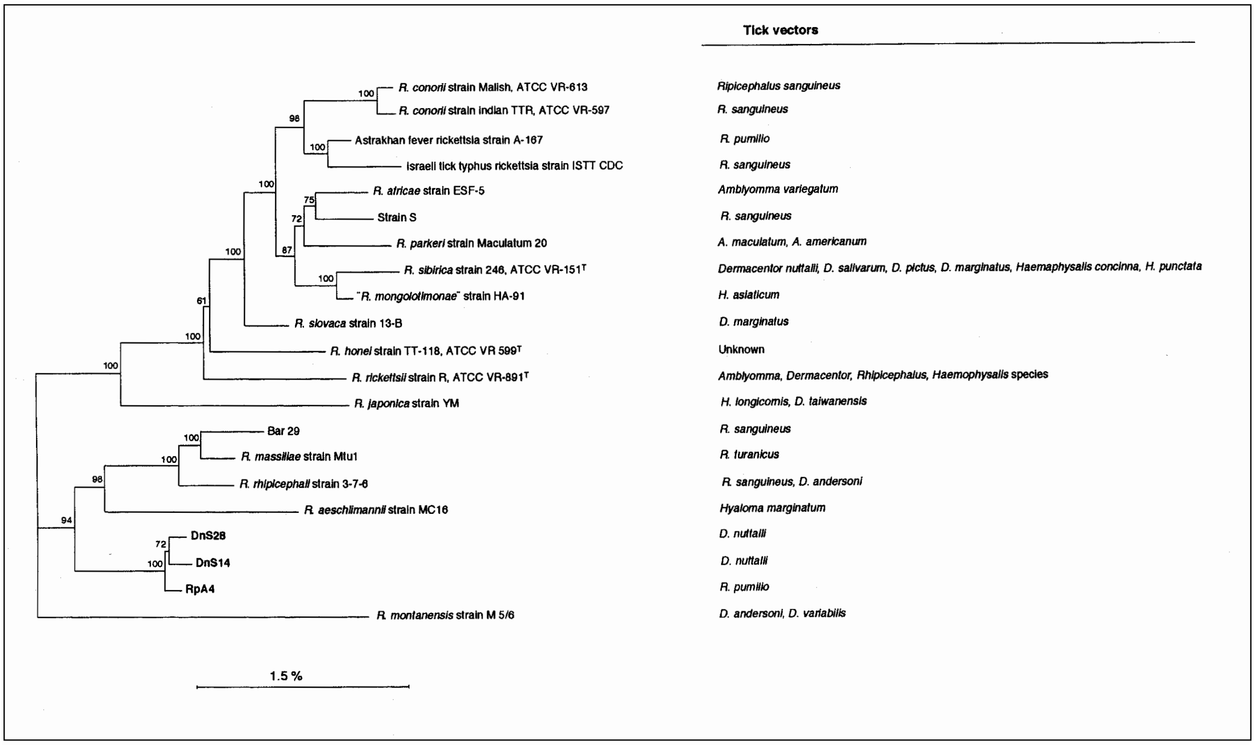

Figure 2.[1] Phylogenetic tree of *Rickettsiae* inferred from comparison of the *ompA* sequences. The known tick vectors for the bacteria presented on the dendrogram are indicated on the right. The *ompA* sequences were aligned by the multisequence alignment program CLUSTAL in the BISANCE software package. Phylogenetic relationships were inferred by using version 3.4 of the PHYLIP software package. Evolutionary distance matrices, generated by DNADIST, were determined by the Kimura method. Matrices were used to construct dendrograms by the neighbor-joining method. Data were also examined by using parsimony analysis (DNAPARS in PHYLIP), and bootstrap analyses were performed to investigate the stability of the trees obtained. Bootstrap values were obtained for a consensus tree based on 100 randomly generated trees by using SEQBOOT and CONSENSE in the same package. The percentage of similarity between strains was determined by using the PC/GENE software package.

[1]The received sequences of the new rickettsial agents have been deposited in GenBank. Sequences of *ompA* were deposited as two parts under accession numbers: DnS28, AF120018 and AF120019; DnS14, AF120021, and AF120020; RpA4, AF120022, and AF120023. The 16S rRNA gene and *gltA* sequences have been deposited in GenBank under accession numbers DnS28, *gltA* - AF120027, 16S rRNA gene – AF120024; DnS14, *gltA* – AF120028, 16S rRNA gene – AF120025; RpA4, *gltA* - AF120029, 16S rRNA gene – AF120026.

agents are close to each other (from 99.7% to 99.9%) and could constitute a new rickettsial species.

In the United States, various tickborne *Rickettsiae* occur in areas endemic for *R. rickettsii,* the agent of Rocky Mountain spotted fever (7). Similarly, in Mediterranean countries, several recently described *Rickettsiae* have been found in ticks of the *Rh. sanguineus* complex in the regions endemic for Mediterranean spotted fever caused by *R. conorii* (6). The effects of the presence of different *Rickettsiae* on the prevalence of infection rates of ticks with individual *Rickettsiae* and on the epidemiology of infections in humans have yet to be determined. *R. sibirica* and the Astrakhan fever agent are prevalent in Siberia and the Astrakhan region, respectively, but the pathogenicity of the new rickettsial genotypes has yet to be investigated.

### Acknowledgments

We thank P.J. Kelly and E. Birtles for reviewing the article.

Dr. Rydkina is a senior researcher at the Gamalaya Institute in Moscow. She was trained as a postdoctoral fellow in Marseille, France. Her main interest is in rickettsial diseases.

### References

1. Raoult D, Roux V. Rickettsioses as paradigms of new or emerging infectious diseases. Clin Microbiol Rev 1997;10:694-719.
2. Roux V, Rydkina E, Eremeeva M, Raoult D. Citrate synthase gene comparison, a new tool for phylogenetic analysis, and its application for the *Rickettsiae*. Int J Syst Bacteriol 1997;47:252-61.
3. Fournier P-E, Roux V, Raoult D. Phylogenetic analysis of spotted fever group rickettsiae by study of the outer surface protein rOmpA. Int J Syst Bacteriol 1998;48:839-49.
4. Roux V, Raoult D. Phylogenetic analysis of the genus *Rickettsia* by 16S rDNA sequencing. Res Microbiol 1995;146:385-96.
5. Billings AN, Teltow GJ, Weaver SC, Walker DH. Molecular characterization of a novel *Rickettsia* species from *Ixodes scapularis* in Texas. Emerg Infect Dis 1998;4:305-9.
6. Marquez FJ, Muniain MA, Soriguer RC, Izquierdo G, Rodriguez-Baño J, Borobio M. Genotypic identification of an undescribed spotted fever group *Rickettsia* in *Ixodes ricinus* from southwestern Spain. Am J Trop Med Hyg 1998;58:570-7.
7. Niebylski ML, Shrumpf ME, Burgdorfer W, Ficher ER, Gage KL, Schwan TG. *Rickettsia peacockii* sp. nov., a new species infecting wood ticks, *Dermacentor andersoni*, in Western Montana. Int J Syst Bacteriol 1997;47:446-52.
8. Weller SJ, Baldridge GD, Munderloh UG, Noda H, Simser J, Kurtti T. Phylogenetic placement of *Rickettsiae* from ticks *Amblyomma americanum* and *Ixodes scapularis*. J Clin Microbiol 1998;36:1305-17.
9. Rudakov NV. Tick-borne rickettsiosis in Russia (epidemiology and current conditions of natural foci). In: Kazar J, Toman R, editors. Rickettsiae and rickettsial diseases. Proceedings of the 5th International Symposium; 1996; Veda, Bratislava. p. 216-20.
10. Eremeeva ME, Beati L, Makarova VA, Fetisova NF, Tarasevich IV, Balayeva NM, et al. Astrakhan fever rickettsiae: antigenic and genotypic analysis of isolates obtained from human and *Rhipicephalus pumilio* ticks. Am J Trop Med Hyg 1994;51:697-706.
11. Kulagin SM, Tarasevich IV, Rubakin PE, Nikitin AM, Krupina ZN. On eradication of Marseilles fever (in Russian). J Microbiol Epidemiol Immunobiol 1960;8:117-21.

